# The Effect of Educational Intervention on Legal Anti-Doping Knowledge and Doping Tendency in Elite Athletes

**DOI:** 10.3390/sports14010035

**Published:** 2026-01-09

**Authors:** Antonela Sinkovic, Dinko Pivalica, Igor Jukic, Miran Pehar, Bozen Pivalica, Ivana Cerkez Zovko, Damir Sekulic

**Affiliations:** 1 Faculty of Kinesiology, University of Zagreb, 10000 Zagreb, Croatia; 2 University Hospital of Split, 21000 Split, Croatia; 3 Faculty of Health Sciences, University of Split, 21000 Split, Croatia; 4 High Performance Sport Center, Croatian Olympic Committee, 10000 Zagreb, Croatia; 5 Faculty of Science and Education, University of Mostar, 88000 Mostar, Bosnia and Herzegovina; 6 High Performance Sport Center, University of Mostar, 88000 Mostar, Bosnia and Herzegovina; 7 Faculty of Kinesiology, University of Split, 21000 Split, Croatia

**Keywords:** health education, randomized controlled trial, doping in sports, logistic models

## Abstract

Studies have rarely examined the effects of changes in legal anti-doping knowledge (LADK) on doping tendencies in athletes. This study aimed to evaluate the effectiveness of a structured educational intervention focused on LADK and to analyze how LADK changes affect elite athletes’ doping tendency. The participants were athletes (*n* = 310; 156 females; 24.1 ± 4.2 years of age), all actively competing at the senior national or international level in either individual (N = 119) or team sports (N = 191), tested on sociodemographic-, sport-, doping-factors (including doping tendency—DT), and LADK. Participants were randomly divided into an experimental group (E: N = 140) and a control group (C: N = 170). The E group participated in a structured educational program on LADK. A pre- and posttest design was used to evaluate changes in LADK (dependent variable). Logistic regression was calculated to evaluate the association between LADK and binarized DT (negative vs. neutral/positive DT). Factorial ANOVA for repeated measurements revealed significant improvement in LADK in the E group, with significant ANOVA effects for time (F test = 35.8, *p* < 0.05) and time × group interaction (F test = 12.27, *p* < 0.05). The logistic regression did not reveal significant correlations between LADK and DT. Further studies exploring younger athletes, as well as long-term, multidimensional interventions, are warranted.

## 1. Introduction

Doping remains one of the most important threats to the integrity of sport, undermining both the fair-play and the health of athletes [1,2]. Global anti-doping efforts, led by the World Anti-Doping Agency (WADA) and enforced through the World Anti-Doping Code (WADC), aim to ensure a level playing field and uphold the core values of clean sport [3]. These efforts are critical not only in identifying violations but also in fostering a sporting culture on the basis of ethics, safety, and respect for rules. The consequences of doping behavior extend beyond the individual athlete, potentially damaging the reputation of teams, nations, and entire sporting disciplines. As a result, some sporting communities are known to be stigmatized with regard to doping [4]. Moreover, there is no doubt that doping presents serious health risks, including cardiovascular, hormonal, and psychological complications [5]. Therefore, sustaining anti-doping efforts requires not only effective testing procedures but also comprehensive education and doping-related literacy in sports societies worldwide.

To support the prevention of doping behavior in sports, substantial global efforts have been invested in anti-doping education, particularly through programs such as WADA’s International Standard for Education, national anti-doping campaigns, and sport-specific educational initiatives [2,6]. These programs aim to promote clean sport values, raise awareness of banned substances, and clarify procedural issues of anti-doping policy. However, the effectiveness of these educational interventions remains contested. Most importantly, data from WADA and national reports suggest that the rate of adverse analytical findings has remained relatively stable over the last 20–30 years [7]. This stagnation raises concerns about whether current educational strategies are sufficiently targeted and comprehensive to alter the doping behavior of athletes. It also highlights the need to evaluate not only whether athletes are informed but also what they are taught.

Since doping behavior is generally observed as a practice that undermines both athletes’ health and fair play, these topics dominate most anti-doping education campaigns [8]. On the other hand, although it is highlighted as one of the crucial facets of anti-doping efforts, the legal dimension of the anti-doping policy framework remains significantly underemphasized [9,10]. In other words, it seems that educational efforts overlook critical legal principles of anti-doping policy, such as strict liability, procedural fairness, and the right to appeal. In the meantime, WADC functions as a binding legal instrument, and athletes are held accountable for its violations regardless of intent or awareness [3]. Therefore, it is logical to expect that the lack of “legal literacy” can lead to unintentional infractions, delayed appeals, or failure to exercise due process rights and ultimately expose athletes to sanctions. As such, understanding the legal framework is a key factor in protecting athletes’ autonomy and promoting procedural justice in sports. To address this gap meaningfully, the first step in evaluating the effectiveness of any legal education intervention is the availability of a valid and reliable measurement tool; without this tool, there is no accurate way to assess baseline knowledge, monitor changes, or determine whether the intended educational impact has been achieved.

Recognizing the absence of validated tools for assessing legal anti-doping knowledge, a previous study developed and tested the original questionnaire designed to evaluate athletes’ understanding of core legal principles embedded in anti-doping regulations [11]. The instrument demonstrated acceptable levels of reliability and validity, offering a standardized means of measuring legal anti-doping knowledge in sport contexts. The application of this measuring tool among professional athletes revealed notable disparities, with male, older, and more competitively successful athletes scoring significantly higher than their female and less experienced peers [11]. Crucially, lower legal knowledge was associated with more permissive attitudes toward doping behavior, particularly among younger and infrequently tested athletes [11].

Concerns about athletes’ limited understanding of antidoping legal frameworks have been raised in several earlier studies, which have helped inform the development of a measurement tool. For example, Viret (2015) emphasized that ignorance of legal obligations increases the risk of procedural violations and undermines the fairness of disciplinary proceedings [12]. More recently, studies by Weber, Patterson, and Blank (2022) and Faros and Shehu (2024) confirmed that legal literacy remains inadequate among both athletes and coaches, compromising their ability to respond appropriately to anti-doping testing procedures and allegations [13,14]. Together, these findings reinforce the structural importance of integrating legal education into antidoping strategies to ensure regulatory compliance, to uphold procedural justice and, most importantly, to protect athletes’ autonomy [9,10].

Although legal anti-doping knowledge primarily reflects athletes’ understanding of obligations, procedures, and potential sanctions, several theoretical perspectives imply that such knowledge may indirectly shape decision-making related to doping behavior itself. Probably, the most important is deterrence theory, originating from classical criminology, which proposes that people are less likely to engage in prohibited behavior when they perceive a high likelihood of detection and believe that consequences will be severe and fairly enforced [15]. This theoretical concept was found to be plausible in different fields [16,17], while in the context of anti-doping, could imply that athletes who understand how testing works, what sanctions apply, and how strictly rules are implemented may become more cautious about using banned substances. Additionally, improved understanding of procedural fairness and athletes’ rights may enhance perceived legitimacy of the anti-doping system. Indeed, there is evidence that athletes’ perceptions of legitimacy are closely tied to fair implementation of anti-doping rules [18,19]. Finally, there is recent evidence that transparent, equitable procedures and clear communication of athletes’ rights should be observed as a key to building trust in the anti-doping system [20]. As a result, better legal literacy could reduce moral disengagement and encourage prosocial adherence to clean sport values. Therefore, examining whether legal literacy can affect doping-related orientations remains warranted.

Despite increasing recognition of the importance of legal knowledge in anti-doping efforts, empirical studies examining the effects of legal anti-doping education remain scarce. To the best of our knowledge, no published studies have systematically evaluated whether structured legal education can enhance athletes’ legal literacy and/or influence their attitudes toward doping. This gap limits our understanding of how legal knowledge contributes to doping-clean sports. Therefore, the present study aimed to evaluate the effectiveness of a structured educational intervention focused on antidoping legal knowledge in senior-level athletes at the high-competitive level. Furthermore, we analyzed the relationships between legal knowledge transfer and changes in athletes’ doping tendencies. Initially, we hypothesized that (i) participation in educational interventions would lead to a significant improvement in legal anti-doping knowledge and that (ii) the eventual improvement in knowledge would be associated with a reduction in doping-positive attitudes among participants.

## 2. Materials and Methods

### 2.1. Participants

The participants in this randomized controlled trial study were elite athletes (N = 310; 156 females; 24.1 ± 4.2 years), all of whom were actively competing at the senior national or international level in either individual or team sports. The sample included athletes from a range of Olympic sports, with 119 athletes involved in individual sports (i.e., athletics, swimming, judo, Olympic sailing) and 191 in team sports (i.e., football, basketball, handball, volleyball, water polo). The participants were invited to take part in the study through collaboration with national sport federations and the Olympic Committee. Prior to participation, all the athletes were informed about the study’s aims, potential benefits, and any associated risks. They provided written informed consent and were assured of anonymity, voluntary participation, and the right to withdraw from the study at any point without explanation.

To be included in the study, participants had to meet the following criteria: (1) being older than 18 years, (2) having an active status as a senior-level athlete, (3) having at least one year of competitive experience at the national level or higher, and (4) having Croatian citizenship. Athletes were stratified by sport type (individual vs. team) and randomly allocated to either the experimental (intervention) or the control group. The final sample comprised 68 individual sport athletes in the control group and 51 in the experimental (intervention) group, along with 102 team sport athletes in the control group and 89 in the experimental group, with no significant difference between control and experimental group in distribution of athletes according to type of sport across study groups (Chi square = 0.41, *p* > 0.05).

This study was approved by the Ethical Committee of the Faculty of Kinesiology, University of Zagreb (approval date: 6 May 2025; reference: 30/2025).

### 2.2. Variables

The study included three main categories of variables: sociodemographic and sport factors, doping-related variables, and legal anti-doping knowledge. Sociodemographic variables consisted of sex (male, female) and age (in years). The sport-related variables included the type of sport (individual vs. team sport), years of active participation in senior-level competition (<5 years, 6–10 years, >10 years), and highest level of sport achievement (national-level competition, national-level medal, European and/or world championship participation/medal, Olympic Games participation/medal).

Doping-related variables were assessed through the following self-report items: athletes’ self-perceived anti-doping knowledge (poor, average, good), their history of anti-doping testing (never, 1–2 times, 3–5 times, >5 times), their opinion about the main problem of doping (doping is mainly a health hazard; doping is against fair play; doping is not a problem), the number of times they were in a situation to participate in antidoping education organized by legal sport associations (No, Rarely, Frequently), the main source of information on doping (coach, medical staff, nutritionist, friend/teammate, formal education/schooling, nonformal education), personal trust with regard to doping issues (coach, medical staff, nutritionist, friend/teammate, I trust no one), and their likelihood of using doping (No way!; I do not know/not sure; I could consider it if there is no health hazard; I could consider it). For subsequent analyses, responses to the likelihood of doping were dichotomized into “negative doping tendency” (first response) and “neutral/positive doping tendency” (all other responses).

Legal anti-doping knowledge was assessed via the previously validated tool measuring the understanding of key legal aspects of the World Anti-Doping Code. The final version of the questionnaire used in this study consisted of 10 true/false items addressing rights, responsibilities, and procedural elements of doping control, with an additional “not sure” option for each item. Specifically, the questionnaire items were as follows: (i) Within one year, an athlete can be tested an unlimited number of times; (ii) the athlete who is in the group designated for testing is obliged to send information about the location for each day in the period of the next 3 months; (iii) an athlete can refuse to give a sample to the organization responsible for testing if he believes that the organization that will test him has political or other prejudices about him (for example, organizations from countries that have political conflicts with the athlete’s country); (iv) the doping control officer must inform the athlete several hours before he will come to test him; (v) the doping control officer can take a urine and blood sample from the athlete; (vi) samples can be stored and reanalyzed over a period of 10 years; (vii) if the analysis of sample B does not confirm the analysis of sample A, the test will be considered negative; (viii) an appeal can be filed against a decision on a violation of anti-doping rules; (ix) if an athlete is found guilty of violating anti-doping rules, that fact will be made public; and (x) an athlete who voluntarily admits to having violated an Each correct answer was scored one point, with total scores ranging from 0 to 10.

All measurement tools were previously used in the local language and have been found to be reliable and valid, while details are available elsewhere [11,21]. Specifically, in the validation study of the legal-anti-doping knowledge questionnaire, the authors demonstrated acceptable test–retest reliability (Cohen’s κ = 0.65) and 84% exact agreement on repeated items. Also, the study reported gender-specific discriminative validity, showing that the instrument distinguished meaningfully between male and female professional athletes [11]. Testing was conducted through the SurveyMonkey digital platform (SurveyMonkey Inc., San Mateo, CA, USA). The participants completed the survey individually, and all the items were presented in the local language (Croatian).

### 2.3. Intervention and Study Protocol

The legal anti-doping education program consisted of 10 structured educational sessions aimed at enhancing participants’ legal understanding of anti-doping regulations and procedures. The curriculum focused on two primary domains: (i) athletes’ rights and obligations under the World Anti-Doping Code and (ii) legal procedures and safeguards involved in the doping control process. The sessions were delivered by a multidisciplinary team that included certified anti-doping officers, sports law experts, and experienced coaches, ensuring a balanced approach that combined theoretical legal content with practical, sport-specific relevance.

To maximize engagement and real-world applicability, the program emphasized experiential learning strategies rather than abstract legal instruction. Each session featured interactive discussions, real-case scenarios, and examples drawn from the instructors’ professional experience in doping control and legal proceedings (note that educators were medical and legal authorities actively involved in anti-doping protocols). The participants were encouraged to actively participate, pose questions, and reflect critically on ethically or procedurally complex situations. The curriculum design was directly informed by prior research on antidoping legal knowledge, particularly studies identifying common knowledge gaps among sport stakeholders in southeastern Europe [11,22]. Special attention was given to procedural nuances in doping control, such as notification, sample collection, documentation requirements, and athletes’ rights during appeals and disciplinary hearings. These elements were deliberately integrated into the program to address the specific areas where legal literacy has been shown to be weakest. The educational materials and structure of the intervention are available upon request from the authors. The characteristics of the educational program are presented in Table 1.

According to WADA’s International Standard for Education, anti-doping education employs both cognitive and affective approaches [3]. Cognitive models focus on delivering information-based programs to enhance knowledge about anti-doping, while affective models aim to instill this knowledge through the development of personal values and ethical principles. These approaches are grounded in different theoretical frameworks (i.e., social learning theory, health belief model, and socio-affective theories), which offer complementary insights into how athletes can be supported in building the self-efficacy needed to resist the use of prohibited substances [2]. Furthermore, preventive interventions also take into account the nature of athlete involvement. Most specifically, participation can be active, where individuals are directly engaged in the learning process, or passive, where they receive information with minimal interaction [2,23].

In our educational approach, we followed the cognitive model by providing information and encouraging reflection on doping-related issues. Also, athletes were actively involved in the program, sharing their personal experiences and expressing their opinions during the educational sessions. Educators deliberately encouraged this active participation in order to frame discussions within specific, relatable educational contexts rather than relying solely on abstract or theoretical concepts.

**Figure 1:**
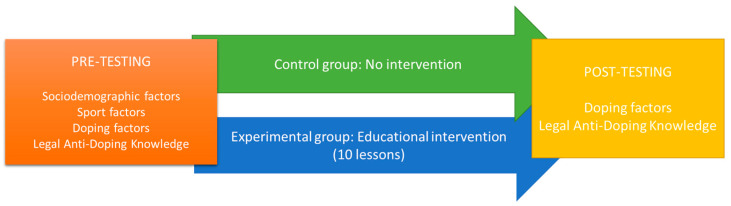
Study protocol.

An overview of the study design and intervention timeline is provided in Figure 1.

### 2.4. Statistics

After the normality of the distributions was assessed, the means and standard deviations were calculated for age and legal anti-doping knowledge scores, whereas the frequencies and percentages were reported as descriptive statistics for the remaining variables.

To identify the differences in categorical and ordinal variables between the control and experimental groups, the chi square (χ^2^) test was performed.

To assess the effects of the intervention on legal anti-doping knowledge, a multifactorial repeated measures analysis of variance (ANOVA) was performed. The model included three fixed (main) factors (effects): Group (experimental vs. control), Time (pretest vs. posttest), and Sport type (individual vs. team). All main effects and the following interaction effects were tested: Group × Time, Group × Sport type, Time × Sport type, and the three-way interaction Group × Time × Sport type. Effect sizes were reported via partial eta squared (η^2^), with values interpreted as follows: η^2^ = 0.01 (small effect), η^2^ = 0.06 (medium effect), and η^2^ ≥ 0.14 (large effect). Where significant interactions were identified, Bonferroni-adjusted post hoc comparisons were conducted.

To evaluate the relationship between legal anti-doping knowledge (predictor) and binarized doping tendency (criterion; negative vs. neutral/positive doping tendency), univariate logistic regression was performed with pre- and post-testing results, separately for the experimental and control groups, with odds ratios (ORs) and 95% confidence intervals (95% CIs) reported.

All analyses were performed via Statistica ver 13.5 (Tibco Inc., Palo Alto, CA, USA), and a *p*-level of 95% was applied.

## 3. Results

Descriptive statistics and differences between the control (C) and experimental (E) groups for the sociodemographic and sport variables analyzed in the study at pre-measurement are presented in Table 2. Most importantly, the majority of the participants had substantial experience in sports, with significant differences between C and E among individual sport athletes (χ^2^ = 6.22, *p* < 0.05). Because of the null frequencies, the differences between groups could not be calculated for the variable of sport success (sports competitive achievement), but it might be said that the C and E groups in both subsamples (individual- and team-sport athletes) had similar sport achievements, while a significant proportion of the studied athletes participated in international competitions.

When observed specifically for athletes involved in individual sports, groups C and E did not differ in the studied doping factors. No significant difference between E and C in doping factors was found for individual sport athletes. Among the team-sport athletes, those in the C group participated in antidoping testing more often than those in the E group did (χ^2^ = 12.83, *p* < 0.05). The majority of the studied athletes considered doping to be a fair-play issue (approximately 60% of all participants), whereas >60% of them reported that they were not familiar with organizing anti-doping education by their sporting authorities. Coaches and medical staff were declared to be the main sources of information on doping in >60% of the studied athletes. Finally, the neutral/positive doping tendency is low, with more than 90% of athletes reporting a negative doping tendency (Table 3).

The results of the multifactorial ANOVA for repeated measurements for legal antidoping knowledge as the dependent variable are presented in Table 4. In brief, the ANOVA main effects were significant for Group (F test = 5.05, *p* < 0.05, small effect) and Time (F test = 35.80, *p* < 0.05, medium effect). The interaction effects were significant for Sport × Group (F test = 8.30, *p* < 0.05, small effect) and Time × Group (F test = 12.27, *p* < 0.05, small effect).

**Figure 2:**
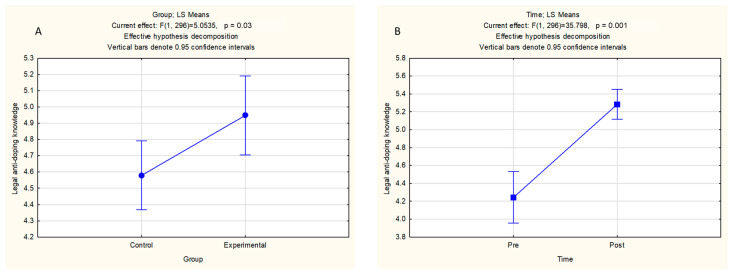
Descriptive statistics for ANOVA main effects for Group—differences between experimental and control group for both measurements (**A**) and Time—differences between pre- and post-measurement for both groups (**B**).

The E group achieved better results in terms of legal anti-doping knowledge than the control group did (4.94 ± 0.12 and 4.57 ± 0.10 for the E and C groups, respectively) (Figure 2A). The anti-doping legal knowledge in the total sample was greater at post- than at pretest (4.24 ± 0.15 vs. 5.28 ± 0.08 for pre- and post-measurement, respectively) (Figure 2B).

**Figure 3:**
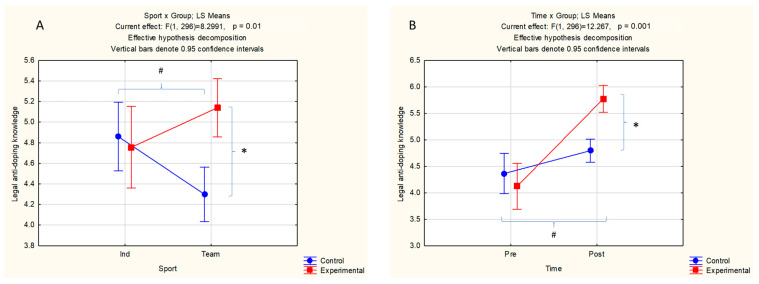
Descriptive statistics and significant post hoc differences for ANOVA interaction effects for sport × group for both measurements ((**A**): * *p* < 0.05 within the team sport group, ^#^ *p* < 0.05 within the control group) and time × group for both type of sports ((**B**): * *p* < 0.05 between groups at post-measurement, ^#^ *p* < 0.05 between measurements for the experimental group).

Significant ANOVA interaction effects with post hoc differences are presented in Figure 2. Significant post hoc differences were found between athletes involved in individual and team sports (for C group only), with better results in legal antidoping testing in individual sport athletes (4.85 ± 0.16 vs. 4.29 ± 0.13). Additionally, team-sport athletes from the E group achieved better results than E group team sport athletes (5.14 ± 0.14 and 4.30 ± 0.13 for E and C, respectively) (Figure 3A). The E group significantly improved their legal anti-doping knowledge during the study course (from 4.12 ± 0.22 to 5.77 ± 0.13) (Figure 2B). Finally, C and E differed significantly in the dependent variable at posttest, with better knowledge in the E group (4.70 ± 0.11 and 5.77 ± 0.13 in C and E, respectively) (Figure 3B).

When legal anti-doping knowledge was correlated with a binarized doping tendency (negative vs. neutral/positive doping tendency), no significant associations were found for the E group (OR = 1.25, 95% CI: 0.90–1.73, *p* = 0.17) or for the C group (OR = 0.95, 95% CI: 0.76–1.19, *p* = 0.70) in the pretest. Similarly, correlations did not reach statistical significance in the posttest data (OR = 0.79, 95% CI: 0.55–1.29; OR = 1.35, 95% CI = 0.80–2.29 for C and E, respectively).

## 4. Discussion

This study aimed to evaluate the effects of a specific educational program on changes in legal anti-doping knowledge in elite athletes from Croatia. The results revealed several important findings. First, educational intervention improved the participants’ legal anti-doping knowledge, with a more evident effect on those athletes involved in team sports. Second, increased knowledge was not reflected in changes in doping tendency. Therefore, our first study hypothesis can be accepted, whereas the second hypothesis should be rejected.

### 4.1. The Improvement in Antidoping Legal Knowledge as a Result of Education

A significant improvement in athletes’ legal anti-doping knowledge as a result of the educational intervention was expected. Specifically, this result mirrors our recent findings in athlete support personnel (ASP), where coaches and medical professionals who underwent similar educational programs also achieved substantial gains in legal anti-doping knowledge [22]. Importantly, in both studies, the participants initially demonstrated a limited understanding of key legal concepts, including strict liability, procedural rights, and the scope of anti-doping sanctions. When the initial results of both studies were compared, the low level of knowledge was even more evident in the athletes studied here than in those previously studied with the ASP. This is mostly a result of relatively good knowledge among medical professionals, whose greater knowledge logically increased the average score for all ASPs [22]. For the current study, it is important to note that such a baseline deficit provided considerable room for educational impact, consequently resulting in significant improvement in legal knowledge among athletes. However, another issue that deserves attention is the significant improvement in athletes’ knowledge.

The authors are convinced that a key factor contributing to the success of the intervention was the application of experiential learning principles, which are initially considered appropriate in the context of our study (i.e., adult education in professional sporting contexts). As presented in the Methods section, the education was led by certified anti-doping officers and legal experts. They grounded the legal anti-doping content in real-world examples drawn from actual disciplinary cases, doping control scenarios, and appeal processes. Initially, we tried to develop the education process on the basis of Kolb’s experiential learning theory, which posits that knowledge is best acquired through a cycle of concrete experience, reflection, conceptualization, and active experimentation [24].

In more detail, it is generally accepted that courses designed around experiential learning increase student engagement and motivation, which are the issues we were specifically interested in this experiment [25]. Additionally, this type of learning has been shown to be highly effective in education characterized by limited time, which was one of the greatest challenges in our study [26]. Therefore, initially we believed that the effectiveness of the educational program would be improved if education included the anti-doping legal concepts within authentic sport situations. After being actively involved in the whole process, the authors of the study may witness that the participating athletes were not merely passive recipients of educational material. In contrast, they were actively engaged, asked questions, and applied legal frameworks to realistic scenarios, whereas athletes who had experience with anti-doping tests presented their own experiences and actively discussed some specific issues together with other athletes and educators. As a result, educational intervention was informative, dynamic, and evidently effective. Supportively, studies repeatedly report better outcomes of the educational interventions where students were actively involved in learning, which was explained by increased student participation and engagement, but also by better perception of the studied problem in more active students from different fields [27,28].

The greater improvement observed among team sport athletes than among their individual sport peers can largely be attributed to differences in pretesting legal knowledge. Specifically, team sport athletes began the intervention with significantly lower pretest scores than their colleagues involved in individual sports did (see Results for more details). Moreover, lower knowledge (or ability) generally leads to greater improvement in the studied capacity [29]. Therefore, the initial difference in legal anti-doping knowledge between team and individual sports provided greater scope for educational gains in the former ones.

However, although the subgroup of team-sport athletes showed more pronounced improvement across the intervention period, the posttest scores did not significantly differ between team- and individual-athletes. This indicates that the educational program actually closed the initial knowledge gap. In this context, we highlight that the more evident gains among team athletes do not reflect superior learning capacity but rather a more substantial need for targeted legal education. Even a recent study on athlete support personnel revealed more evident improvements in legal anti-doping knowledge among coaches than among medical personnel, while the latter possessed better knowledge during pretesting [22].

We may say that initial differences in legal knowledge between team athletes and individual sport athletes were expected. Specifically, previous studies addressing general anti-doping knowledge have frequently revealed better knowledge among athletes involved in individual sports than among their team-sport peers [21,30,31]. From the perspective of antidoping (legal) knowledge, it can be understood through two interrelated factors. First, the anti-doping testing frequency tends to be higher in individual sports, which is supported even herein (please see Results for details). This approach almost certainly increased their knowledge of relevant legal content because this repeated procedural engagement undoubtedly serves as a learning mechanism. It logically increased athletes’ familiarity with legal issues even if they were not involved in some type of formal education.

Second, our results indicate that coaches (and medical staff) are the main sources of information on doping for athletes, which is consistent with previous research [32]. Moreover, athlete-support personnel dynamics in individual sports are typically more intimate and continuous than those in team sports. In individual sports, athletes often rely on a small, consistent team of coaches and medical staff. On the other hand, the support structures in team sports are broader and somewhat “scattered” across different compartments. Therefore, interactions between team-sport athletes and coaches and medics may be less personal.

Supportively, previous research has shown that individual athletes often receive more direct guidance on doping-related matters from their support teams, which is connected to a higher prevalence of doping in individual sports [33,34]. Although we could not find direct empirical evidence on this topic, this fact can naturally be translated to legal anti-doping knowledge, resulting in higher baseline legal literacy among individual sport athletes. Therefore, the weaker support network integration in team sports may partially explain both (i) the lower initial scores and (ii) the better responsiveness to structured legal education we have achieved.

In addition, variations in doping-test frequency may contribute to the observed differences in legal knowledge between types of sports. Namely, athletes involved in individual sports were evidently tested more frequently than their peers involved in team sports. This could be a result of the performance-based qualification systems and individualized tracking protocols, which altogether, provide them with more exposure to procedural elements of anti-doping control, logically resulting in their better legal knowledge. In contrast, team-sport athletes observed herein experienced less frequent testing, which is probably a result of collective testing policies in these sports [35]. This logically reduces their opportunity to learn specific anti-doping rules and procedures in “real-world” settings.

Finally, cultural and normative influences may also play a role. Team sports are highly group-oriented, and shared values can reduce individual risk-taking by emphasizing collective responsibility and public scrutiny within the team environment [36]. Conversely, individual sports tend to foster a stronger performance-focused culture where personal success is observed as an “achievement goal”. This altogether can result in higher motivation to seek any kind of advancement (including doping). However, the logical consequence of such higher motivation is the necessity to understand rules associated with it (in our case, anti-doping legal knowledge). These cultural differences between individual and team sports could shape athletes’ baseline awareness of anti-doping regulations and modulate athletes’ responsiveness to the educational program evaluated in this study.

### 4.2. Lack of Correlation Between Legal Anti-Doping Knowledge and Doping Tendencies

Although we hypothesized that improved knowledge of antidoping legal knowledge would be correlated with a lower likelihood of doping, this did not happen, as we did not find any correlation between legal knowledge and doping tendency. There are several possible explanations for such findings. First, it can be partly related to the fact that the study sample consisted of adult, advanced-level athletes. At this stage in sporting careers, most of the behavioral tendencies (i.e., those associated with ethical issues and decisions, those related to specific career-related risks) are established and resistant to change. Research suggests that adult populations tend to exhibit greater attitudinal stability than adolescents or younger people do, especially in regard to deeply internalized beliefs or “value-driven behaviors” [37].

Overall, the evidence consistently supports the idea that attitudes become more stable with age across various domains, including social group attitudes, risk-taking, and values, all of which are clearly connected to doping attitudes among athletes. This increased stability is likely due to the formation of attitudes early in life and a decline in susceptibility to change as individuals grow older [38]. In addition, most (if not all) of the athletes involved in the study are already embedded in structured, competitive environments with clear expectations and routines. This is simply evident from their competitive achievements (please see Results for details). This could further reinforce their relative rigidity and explain why the evident improvement in antidoping legal knowledge was not related to their doping tendency.

Apart from the previously discussed “attitudinal stability”, another important issue limiting the detection of significance of correlation is related to a specific “statistical issue”. Our initial results clearly indicated a small proportion of athletes who reported a neutral/positive attitude toward doping at study baseline (<10% of athletes). This low prevalence limits the potential for measurable changes in this variable over time. Statistically, this creates a “floor effect,” where responses cluster at the end of the scale, leaving little room for further decrease [39]. Although this low prevalence of neutral/positive doping tendencies could reflect genuine anti-doping convictions, it is also possible that social desirability influences the initial results. Specifically, doping is generally stigmatized in sporting communities [40,41]. Therefore, some participants could respond in a socially acceptable manner and therefore report negative doping tendencies despite their true opinions. Irrespective of the background, the lack of variability at pretest reduces the sensitivity of the measure to capture correlations.

From this perspective, authors are convinced that the composition of the educational team likely reinforced the effects of social desirability bias during the intervention. As previously mentioned, the sessions were delivered by certified anti-doping agents and legal professionals, some of whom were directly affiliated with the national anti-doping organization. While their expertise undoubtedly enhanced the quality of education, their authoritative roles may have also heightened participants’ sensitivity to judgment and evaluation. In such settings, athletes may have been unwilling to disclose permissive attitudes toward doping, even in an anonymous testing environment. In other words, they could have been concerned that their responses could be associated with them personally. If we take into account that all participants were advanced-level athletes, the problem is even more likely because of the high reputational risk [42].

Taken together, these considerations indicate that the absence of a detected relationship between improved legal knowledge and doping tendency in the present study should not be interpreted as evidence of a true lack of association. Rather, the findings are more plausibly explained by methodological constraints, including the mature competitive status of the sample, restricted variability and floor effects in attitudes, and the potential influence of social desirability on self-reported outcomes. Under such circumstances, even meaningful attitudinal changes may remain statistically undetectable. Therefore, our results should be viewed as context-dependent, highlighting the need for future research to incorporate more sensitive attitudinal measures, recruit younger or more heterogeneous samples, and implement longer-term or multi-component interventions. Such improvements will be essential to adequately test whether enhanced legal literacy can ultimately contribute to ethically grounded decision-making and reduce positive doping tendency.

Irrespective of the previous explanations, our findings of the lack of transfer of the legal knowledge on changes in doping tendency actually align with evidence from the recent systematic review which reported that although cognitive (knowledge-based) and affective/values-based anti-doping programs often yield short-term reductions in doping intention and behavior, their effects on deep-seated moral behavior remain negligible [2]. This suggests that while knowledge dissemination is necessary, it is likely insufficient on its own to induce lasting value-driven behavioral change. Most specifically, it is probable that interventions focused solely on information transfer may temporarily shift attitudes or intentions, but not the underlying moral compass or doping tendency.

### 4.3. Limitations and Strengths

This study has several limitations that should be acknowledged. First, the duration of the intervention was relatively short, consisting of only 10 sessions, which may have limited its potential to produce more evident changes in doping attitudes. Additionally, the sample was composed exclusively of senior-level athletes, whose cognitive frameworks and ethical positions are probably well formed. This fact makes attitudinal change through educational intervention inherently more difficult. Therefore, the generalizability of the findings may be limited, especially when considering younger or less experienced athletes, who may be more open to shifts in their attitudes. Third, the same individuals who delivered the educational content were also subjected to pre- and postintervention testing. As discussed, this could have introduced social desirability bias or unintentional influence on how participants approached the assessments. Finally, the doping tendency in this study was evaluated by a dichotomous variable (negative vs. neutral/positive tendency), which could result in non-sensitivity of the criterion. Therefore, in future studies, more sensitive variables examining the doping tendency should be used.

Moreover, to the best of our knowledge, this is one of the first studies to evaluate the effects of a structured legal anti-doping education intervention directly among elite athletes. Second, the sample consisted of high-level senior athletes actively competing at the national and international levels, which enhances the ecological validity of the findings. In other words, the sample of athletes studied ensures the relevance of the results to real-world sport settings. Third, the study employed a validated, athlete-specific tool for assessing legal anti-doping knowledge, allowing for accurate, reliable measurement and meaningful interpretation of educational outcomes. Therefore, we believe that although not the last word on a topic, the study will improve the body of knowledge and initiate further research in this field.

## 5. Conclusions

The present study demonstrated that a structured legal anti-doping education program can successfully improve athletes’ legal literacy, particularly among those who initially displayed lower knowledge levels. These findings highlight the effectiveness of experiential, context-driven education in addressing procedural knowledge gaps.

However, improvements in legal knowledge were not accompanied by measurable changes in doping tendency. Importantly, these null findings should be interpreted cautiously due to methodological characteristics of our study (i.e., highly experienced athlete sample, the strong social stigma surrounding doping, and clear indications of floor effects in attitudinal measures). It is altogether likely to limit the detection of subtle changes in value-based outcomes. Therefore, the absence of a statistical association does not imply that legal knowledge is irrelevant to behavior, but rather underscores the complexity of factors associated with doping tendency in advanced-level athletes.

Future educational programs should consider incorporating longer education and samples more open to attitudinal change (e.g., youth athletes) to better evaluate whether enhanced legal literacy can change attitudes toward doping. Additionally, interventions targeting ethical reasoning, motivational factors, and broader sociocultural influences are warranted.

## Figures and Tables

**Table 1:** Overview of the Legal Anti-Doping Education Intervention.

Component	Description
Target audience	Senior-level elite athletes (national and international competitors)
Number of sessions	10 structured educational sessions (10 × 45 min)
Delivery team	Certified anti-doping officers, sports law experts, and experienced coaches (medical and legal authorities involved in anti-doping processes)
Pedagogical approach	Experiential learning: interactive discussions, real-case scenarios, practical examples from testing and legal proceedings
Learning engagement	Active participation encouraged: athletes asked to share personal experiences, ask questions, and reflect on procedural or ethical dilemmas
Theoretical foundation	Cognitive approach aligned with WADA International Standard for Education; complemented by socio-affective elements (reflection and discussion)
Content emphasis	Procedural nuances: notification, sample collection, documentation requirements, appeals, and disciplinary processes
Evidence-based design	Curriculum informed by prior research identifying knowledge gaps among sport stakeholders in Southeastern Europe
Relevance	Sport-specific contextualization to enhance applicability and support informed legal decision-making
Materials availability	Full content and educational materials available upon request from the authors

**Table 2:** Descriptive statistics for sociodemographic and sport factors (F—frequencies, %—percentages) with differences between the control (C) and experimental (E) groups (Chi square—χ^2^).

Variables	Individual Sports			Team Sports		
	C *n* = 68	E *n* = 51	χ^2^	C *n* = 102	E *n* = 89	χ^2^
	F (%)	F (%)	χ^2^ (*p*)	F (%)	F (%)	χ^2^ (*p*)
Gender			0.07 (0.79)			0.08 (0.77)
Male	37 (54.4)	29 (56.9)		48 (47.1)	40 (44.9)	
Female	31 (45.6)	22 (43.1)		54 (52.9)	49 (55.1)	
Missing	0 (0)	0 (0)		0 (0)	0 (0)	
Experience in sport			6.22 (0.04)			1.63 (0.44)
<5 years	14 (20.6)	12 (23.5)		32 (31.4)	27 (30.2)	
6–10 years	21 (30.9)	26 (51)		36 (35.3)	26 (29.4)	
>10 years	31 (45.6)	13 (25.5)		32 (31.4)	36 (40.4)	
Missing	1 (1.5)	0 (0)		2 (2)	0 (0)	
Sport success			NC			NC
National level competition	14 (20.6)	12 (23.5)		30 (29.4)	36 (40.4)	
National level achievement (medal)	35 (51.5)	17 (33.3)		40 (39.2)	32 (36)	
European and/or World Championship (participation and/or medal)	18 (26.5)	21 (41.2)		31 (30.4)	16 (18)	
Olympic Games (participation and/or medal)	1 (1.5)	0 (0)		0 (0)	5 (5.6)	
Missing	1 (1.5)	0 (0)		1 (1)	1 (1.1)	

Note: Missing values were not included in the calculation of the χ^2^ test; NC—χ^2^ test was not calculated because of null frequencies.

**Table 3:** Descriptive statistics for doping-related factors (F—frequencies, %—percentages) with differences between the control (C) and experimental (E) groups (Chi square—χ^2^).

Variables	Individual Sports			Team Sports		
	C *n* = 68	E *n* = 51	χ^2^	C *n* = 102	E *n* = 89	χ^2^
	F (%)	F (%)	χ^2^ (*p*)	F (%)	F (%)	χ^2^ (*p*)
Self-opinion about personal doping knowledge			0.67 (0.71)			4.75 (0.09)
Poor	36 (52.9)	29 (56.9)		62 (60.8)	51 (57.3)	
Average	14 (20.6)	12 (23.5)		24 (23.5)	16 (18)	
Good	16 (23.5)	9 (17.6)		11 (10.8)	20 (22.5)	
Missing	2 (2.9)	1 (2)		5 (4.9)	2 (2.2)	
Participation in anti-doping testing			1.56 (0.45)			12.83 (0.001)
Never						
1–2 times	39 (57.4)	27 (52.9)		69 (67.6)	58 (65.2)	
3–5 times	12 (17.6)	14 (27.5)		20 (19.6)	10 (11.2)	
>5 times	13 (19.1)	8 (15.7)		3 (2.9)	16 (18)	
Missing	4 (5.9)	2 (3.9)		9 (8.8)	6 (6.7)	
The main problem of doping						NC
Doping is health hazard	18 (26.5)	11 (21.6)		32 (31.4)	36 (40.4)	
Doping is against fair-play	48 (70.6)	35 (68.6)		61 (59.8)	44 (49.4)	
I do not see doping as a problem at all	1 (1.5)	0 (0)		0 (0)	7 (7.9)	
Missing	1 (1.5)	5 (9.8)		9 (8.8)	3 (3.4)	
Organization of the anti-doping education (by Team, Federation, etc.)			0.83 (0.65)			5.99 (0.06)
No, I cannot remember that it was organized	38 (55.9)	32 (62.7)		62 (60.8)	61 (68.5)	
Yes, but rarely	26 (38.2)	16 (31.4)		27 (26.5)	27 (30.3)	
Yes, often	4 (5.9)	2 (3.9)		9 (8.8)	1 (1.1)	
Missing	0 (0)	1 (2)		3 (3)	0 (0)	
The main source of information on doping						NC
Coach	26 (38.2)	21 (41.2)		21 (20.6)	22 (24.7)	
Medical staff	21 (30.9)	13 (25.5)		35 (34.3)	19 (21.3)	
Nutritionist	2 (2.9)	1 (2)		1 (1)	4 (4.5)	
Friend, teammate, etc.	5 (7.4)	5 (9.8)		23 (22.5)	21 (23.6)	
Formal education	1 (1.5)	0 (0)		3 (2.9)	0 (0)	
Nonformal education	10 (14.7)	6 (11.8)		11 (10.8)	14 (15.5)	
Missing	4 (5.9)	5 (9.8)		9 (8.8)	9 (10.1)	
Personal trust with regard to doping			0.78 (0.94)			1.43 (0.83)
Coach	22 (32.4)	16 (31.4)		36 (35.3)	30 (33.7)	
Medical staff	28 (41.2)	20 (39.2)		37 (36.3)	34 (38.2)	
Nutritionist	1 (1.5)	2 (3.9)		1 (1)	2 (2.2)	
Friend, teammate, etc.	7 (10.3)	6 (11.8)		17 (16.7)	16 (18)	
No one	4 (5.9)	3 (5.9)		2 (2)	4 (4.5)	
Missing	5 (7.4)	4 (7.8)		9 (8.8)	2 (2.2)	
Likelihood of doping behavior			NC			NC
No way!	61 (89.7)	45 (88.2)		96 (94.1)	84 (94.4)	
Do not know (not sure)	4 (5.9)	2 (3.9)		2 (2)	3 (3.4)	
I’d consider if there will be no health hazard	1 (1.5)	2 (3.9)		2 (2)	2 (2.2)	
I’d consider	0 (0)	1 (2)		0 (0)	0 (0)	
Missing	2 (2.9)	2 (3.9)		1 (1)	1 (1.1)	

Note: Missing values were not included in the calculation of the χ^2^ test; NC—χ^2^ test was not calculated because of null frequencies.

**Table 4:** Main and interaction effects of the multifactorial ANOVA for repeated measurements, and effect size (η^2^).

ANOVA Effects	SS	df	MS	F Test	*p*	η^2^
Intercept	12,331.06	1	12,331.06	3372.32	0.001	0.92
Main effect: Sport (Individual vs. Team)	1.05	1	1.05	0.29	0.593	0.00
Main effect: Group (Intervention vs. Control)	18.48	1	18.48	5.05	0.025	0.02
Interaction effect: Sport × Group	30.35	1	30.35	8.30	0.004	0.03
Error	1082.34	296	3.66			
Main effect: Time (Pre- to Post-measurement)	147.26	1	147.26	35.80	0.001	0.11
Interaction effect: Time × Sport	9.85	1	9.85	2.39	0.123	0.01
Interaction effect: Time × Group	50.46	1	50.46	12.27	0.001	0.04
Interaction effect: Time × Sport × Group	4.47	1	4.47	1.09	0.298	0.00
Error	1217.59	296	4.11			

Note: SS—sum of squares, df—degrees of freedom, MS—mean square.

## Data Availability

Data will be provided to all interested parties upon reasonable request.
